# Reflections on Small-Class Teaching in Veterinary Medicine Undergraduate Programs in China

**DOI:** 10.3390/vetsci11090432

**Published:** 2024-09-13

**Authors:** Pengpeng Xia, Ziyue Chen, Yi Luo, Xiangyu Li, Xin Ma, Siqi Lian

**Affiliations:** 1 College of Veterinary Medicine, Institute of Comparative Medicine, Yangzhou University, Yangzhou 225009, China; chenziyue1122@yeah.net (Z.C.); luoyi_0910@163.com (Y.L.); lxy2419216465@163.com (X.L.); nn20000413@163.com (X.M.); mx120180715@yzu.edu.cn (S.L.); 2 Jiangsu Co-Innovation Center for Prevention and Control of Important Animal Infectious Diseases and Zoonoses, Yangzhou 225009, China

**Keywords:** top-notch innovative talents, higher education, veterinary education, teaching reform

## Abstract

**Simple Summary:**

This paper presents an initial exploration of the strategy and direction of small-class teaching within veterinary medicine undergraduate programs in China. The aim is to summarize the characteristics, advantages, and existing challenges of this approach as implemented in China, with the goal of fostering a new generation of veterinarians equipped with robust professional knowledge, excellent practical abilities, and strong scientific literacy. It also aims to identify effective strategies for expanding the implementation of small-class teaching in order to cultivate top innovative talents.

**Abstract:**

As a cornerstone of higher education in China, the quality of undergraduate teaching is crucial for nurturing high-caliber innovative talents that meet the needs of national and social development. Small-class teaching has emerged as a focal point in the reform of undergraduate education and represents an important approach to cultivating top-notch innovative talents. Veterinary medicine is a scientific discipline that encompasses the prevention, control, diagnosis, and treatment of animal diseases. It also involves efforts to prevent the transmission of animal diseases to humans. The training of professionals in this field should emphasize the integration of theoretical knowledge with practical skills. Therefore, small-class teaching facilitates open communication between educators and students, which is instrumental in fostering a new generation of veterinarians equipped with robust professional knowledge, excellent practical abilities, and strong scientific literacy. This paper provides a preliminary exploration into the strategy and direction of small-class teaching within China’s veterinary medicine undergraduate programs by summarizing its characteristics, advantages, and existing challenges. The unique traits of veterinary medicine are also taken into consideration during this analysis.

## 1. Introduction

Small-class teaching aims to reduce class size, control the number of students (usually 20–30 people), innovate teaching content, methods, and classroom organization forms, and enrich teaching resources to enhance interaction between teachers and students as well as among students themselves [1]. This creates a positive feedback mechanism that ultimately improves the quality of education. The goal of these teaching reforms is to cultivate top innovative talents and address the problems associated with opening courses that are lacking in quality, infrastructure, or personnel. Most importantly, small-class teaching addresses the drawbacks of prioritizing quantity over quality in teaching and avoids training only a few competent professionals.

Compared with universities in Europe and the United States, small-class teaching for undergraduate students in China started relatively late, and its overall implementation is not yet widespread [2]. Currently, most small-class teaching still relies heavily on teacher lectures and demonstrations. While concepts such as flipped classrooms [3] and research-based teaching [4] are often utilized, they do not always achieve their intended impact. It should be noted that simply replicating educational models from Europe or the United States may not be suitable for Chinese student training due to issues such as imbalanced teacher–student ratios, low student participation rates, and outdated teaching methods prevalent in several local universities. Therefore, how to effectively implement small-class teaching at the undergraduate level remains a hot topic for exploration within higher education in our country. This paper utilizes a systematic literature review methodology to analyze 28 journal articles or policy documents published between 2013 and 2023. All these sources are accessible from the PubMed and Web of Science databases and government websites. The objective of this study is to analyze the current advancements and limitations of small-class teaching in China and to identify effective strategies for expanding the implementation of this pedagogical approach to cultivate talents in other countries and regions.

## 2. The Importance of Implementing Small-Class Teaching

In March 2012, the Ministry of Education issued “Several Opinions on Comprehensively Improving the Quality of Higher Education” (hereinafter referred to as the “Opinions”). The document proposed to reform the talent training mode of higher education by implementing a tutorial system, encouraging small-class teaching, and stimulating students’ learning initiative, enthusiasm, and creativity to cultivate top-notch innovative talents [5]. The Outline of the “National Medium and Long-term Education Reform and Development Plan (2010–2020)” also explicitly states that it is necessary to “deepen the reform of curriculum and teaching methods and promote small class teaching” [6]. Since 2012, Beijing University, Sichuan University, the University of Science and Technology of China, and Xiamen University have successively adopted a teaching model consisting of “large-class lectures” followed by small-class discussions [7], which has effectively improved the quality of talent training. It has been proved that the “small-class teaching” mode is beneficial for stimulating students’ learning initiative, creativity, and inner potential [2]. This makes it an important form of teaching organization worthy of widespread adoption in practice. In 2015, China’s General Office of State Council issued the “Implementation Opinions on Deepening Innovation and Entrepreneurship Education Reform in Colleges and Universities” stating that colleges will expand small-class teaching to eliminate “high scores but low abilities”, focusing instead on cultivating students’ critical and creative thinking skills [8]. Unfortunately, according to a report released by China’s Ministry of Education in 2020 titled “The 2018 National Report on Teaching Quality in Undergraduate Programs of Regular HEIs’”, there remains insufficient innovation in China’s current teaching models. The average course opening rate for professional courses with small classes (30 people or fewer) at colleges/universities stands at only 29.11% [9].

On 6 July 2023, a series of themed press conferences were held by the Information Office of the State Council on the topic of “Authority Departments Talking Starts”. During these conferences, Deng Chuanhuai, Director of the Policy and Regulations Department of the Ministry of Education, once again emphasized the importance of “optimizing teacher management and resource allocation” in response to the population situation. He highlighted that teacher configuration should adapt to the needs of small classes and personalized teaching [10]. This indicates that the gradual implementation of small-class teaching remains an inevitable trend in the development of education in colleges and universities in China at this stage.

As a cornerstone of higher education in a country, improving the quality of undergraduate education is crucial for cultivating top-notch innovative talents that are essential for national development. Contemporary college students grow up in an era characterized by information explosion, open and diversified ideas, and strong individuality. They require an environment with a robust learning atmosphere to better exert their learning initiative and creativity [11]. Given realistic conditions such as significantly reduced learning time for professional courses, it becomes particularly important to provide students with core professional knowledge so that they can freely explore and study. The focus should be on truly empowering them with skills rather than simply imparting knowledge.

Due to issues such as the continuous expansion of college enrollment and the shortage of teachers, Chinese colleges and universities have been employing large class sizes in undergraduate teaching for many years [2]. This teaching mode relies on student consciousness for classroom management, with a lack of emphasis on standard student behavior. Consequently, students often exhibit low engagement, attention, initiative, and participation in the classroom. Furthermore, this approach fails to fully harness students’ learning potential or stimulate their interest in independent inquiry. Large-class teaching also results in an uneven distribution of educational resources, an overemphasis on theoretical knowledge at the expense of practical application, and insufficient knowledge reserves and teaching experience among some instructors [12]. As a result, teaching efficiency and quality suffer. These challenges hinder students’ comprehension, mastery, and application of knowledge, particularly impacting applied or experimental subjects.

In contrast to large-class teaching, small-class formats such as discussions and seminars have demonstrated positive effects across various areas, including knowledge framework construction, formation of comprehensive knowledge systems, facilitation of multidirectional communication between teachers and students, promotion of subject knowledge flow, and cultivation of independent learning abilities. In particular, small-class teaching significantly enhances attention to course content in experimental subjects while stimulating interest in learning. It also promotes hands-on skills development, as well as collaboration and summary ability, and cultivates strong professional qualities [1,13].

## 3. Advantages and Disadvantages of Small-Class Teaching 

The core of small-class teaching is the student body, with an emphasis on being student-oriented and guided by teachers. By effectively integrating teaching and learning, students can be encouraged to think independently, thus continuously developing their ability to identify and solve problems and ultimately apply what they have learned. The advantages of small-class teaching are evident in several aspects. Firstly, a smaller class size gives teachers more opportunities to get to know and understand each student individually, enabling them to tailor their expectations and concerns accordingly. When students perceive such expectations and concerns, it naturally ignites their enthusiasm for learning and enhances the quality of individual learning. This mode of communication also aligns well with the psychological expectations and behavior patterns of college students in today’s era. Additionally, this approach enables timely feedback on teaching effectiveness, which facilitates adjustments in teaching methods, content, and evaluation systems. Such interaction and promotion are unique features of small-class teaching [2,14,15].

**Figure 1:**
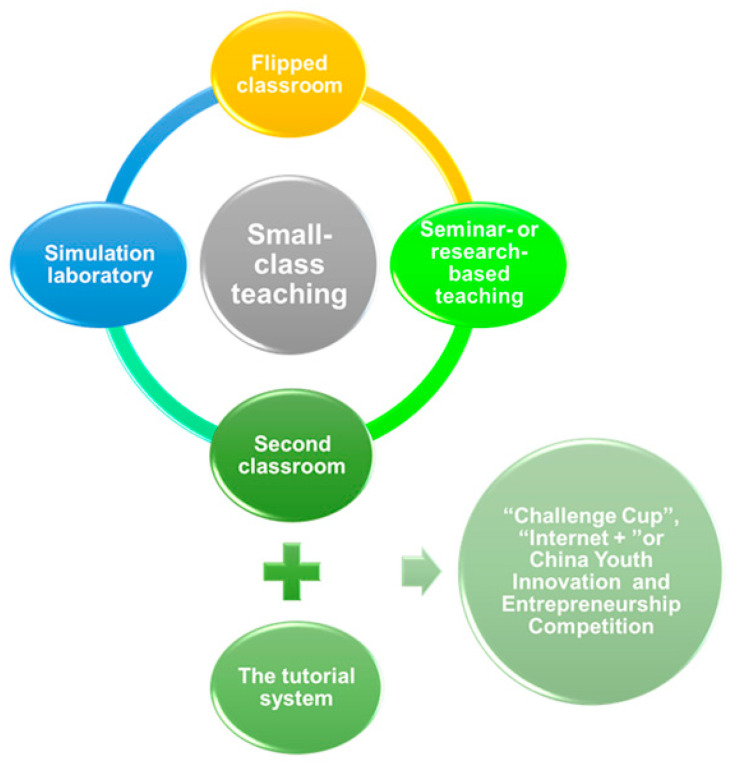
**The instructional approach within the small-class teaching system.** The implementation of the small-class teaching system is supported by new teaching models such as the “simulation laboratory”, “flipped classroom”, “second classroom”, and “seminar” or other research-based teaching methods. Students, under the guidance of a research supervisor, are provided with opportunities to enhance their skills and knowledge through participation in extracurricular academic science and technology competitions such as the “Challenge Cup”, “Internet+”, or the China Youth Innovation and Entrepreneurship Competition. This enables them to further improve and develop in their academic pursuits.

The second aspect involves the successful implementation of new teaching models such as the “simulation laboratory”, “flipped classroom”, and “seminar” (Figure 1). These models facilitate positive interaction between teachers and students, fostering a shared direction within a relaxed and enjoyable classroom atmosphere [3,16]. The development of a “dual-subject” learning model promotes mutual progress for both teachers and students [17], placing equal emphasis on both as the primary participants in the educational process. This approach fosters a collaborative environment where teachers and students can learn from each other, leading to enhanced educational outcomes for all involved. Thirdly, effective integration with teaching reform methods such as the “tutorial system” increases the frequency of teacher–student interaction and extends key knowledge beyond the classroom to extracurricular activities. By leveraging the scientific research resources of mentors, students can engage in targeted scientific research training in laboratories, enhancing their research capabilities. This approach enables personalized education for students while expanding participation in extracurricular academic science and technology competitions such as the “Internet+” Innovation and Entrepreneurship Competition and the “Challenge Cup” National College Student Extracurricular Academic and Technological Works Competition. Furthermore, starting from scientific issues allows for using scientific research to drive teaching practices, with subsequent feedback from teaching results to enhance mentor resources and strengthen scientific research teams. This positive feedback loop ultimately leads to a mutually beneficial cooperation between teachers and students [8,18].

Fourthly, small-class teaching can enhance students’ interest in scientific knowledge, cultivate their belief in, and motivation to contribute to, the revitalization and construction of science and technology in their motherland, and facilitate the realization of their self-value and life pursuits [19]. The case observation and analysis of excellent small-class teaching at Wuhan University demonstrate that learning is not simply a transfer of knowledge but rather a process of active construction by individuals. It integrates “adult” education with “talented” education, emphasizing the cultivation of students’ knowledge, independent learning ability, and moral quality. This approach enables students to learn consciously and proactively through their inner drive, fostering academic aspirations and promoting creative learning [20]. 

Undoubtedly, the comprehensive evaluation of learning and teaching effects in all aspects and throughout the entire process is an indispensable component in the reform of small-class teaching. Currently, one aspect that is often overlooked by both teachers and students in small-class teaching is the use of outdated and singular assessment methods [15]. For instance, different teachers form teaching groups and implement small-class teaching for various classes, yet utilize a uniform scoring system for exams. Despite the exam content falling within the scope of the course outline, different teachers frequently emphasize distinct areas of study which results in varying levels of student mastery over key knowledge points. This discrepancy ultimately manifests itself in differences seen in final exam scores across courses. Such disparities not only diminish enthusiasm among students who actively engage in interactive small-class teachings but also unfairly evaluate teacher effectiveness. Therefore, reducing reliance on final exam results while increasing emphasis on practical application and daily performance can effectively shift students’ long-standing habit of last-minute cramming before exams to foster a more comprehensive evaluation of learning and teaching effects [7].

At a press conference held by the Ministry of Education on 15 September 2022, Wang Hui, Director of the Department of College Students at the Ministry of Education in China, emphasized the importance of continuing to promote educational evaluation reform aimed at “breaking the five only” and innovating talent training models [21]. The meeting underscored that the current evaluation criteria, which are solely based on scores, papers, professional titles, and academic qualifications, are no longer sufficient for the demands of the new era. It is only by advancing the reform concept of “breaking the five only” that we can fundamentally address educational evaluation issues, foster healthy education development, establish a more comprehensive and scientific educational evaluation system, genuinely promote students’ all-round development, and enhance education quality. Small-class teaching is an essential element in the effective implementation of this reform. The implementation of small-class teaching facilitates the integration of multiple teaching methods. Utilizing various forms such as case analysis, role-playing, group presentations, and group discussions for research-based teaching not only enlivens the classroom atmosphere and encourages active student participation but also enhances students’ comprehension and mastery of knowledge. This approach fosters interest, transforms passivity into activity, and stimulates innovative thinking [2,8,15,20]. However, it also places higher demands on teachers’ professional abilities. For instance, effectively addressing students’ questions in a timely manner while providing simple yet comprehensive explanations for their doubts requires exceptional qualities from teachers. Additionally, being able to navigate interdisciplinary knowledge expansion and accurately evaluate student performance are essential skills that outstanding educators must possess. 

However, faculty in higher education institutions are faced with a multitude of pressures related to teaching, scientific research, and personal life. As a result, it is challenging for them to independently develop all the necessary components for small-class construction. Therefore, small-class teaching should also support the revision of faculty assessment criteria, encourage educators to adapt their teaching philosophies and methodologies, actively enhance their instructional capabilities, promptly document their experiences, collaborate as a cohesive unit, and continuously refine the mode and methods of instruction [1,22]. Only through these efforts can we align with national standards for high-quality undergraduate education and genuinely contribute to the cultivation of innovative talents.

## 4. Small-Class Teaching in Veterinary Undergraduate Education

As a predominantly agricultural nation, China is currently undergoing a critical period of modern agricultural transformation [23]. There is an urgent need to expedite the development of high-level, internationally oriented innovative agricultural talents. In the field of veterinary medicine, which is a research-based agricultural science, the curriculum primarily consists of theoretical and experimental courses (practical courses) (Table 1). In large-class settings, it can be challenging for teachers to effectively manage classroom instruction while also ensuring comprehensive knowledge dissemination due to the sheer number of students. Additionally, some students may prioritize participation in extracurricular activities over academic pursuits and settle for merely “passing the test”. However, given the nature of research (or experimental) subjects themselves, inadequate comprehension of theoretical knowledge often results in knowledge gaps that hinder effective application during practical classes. This diminishes learning efficiency and leads to repetition, creating a detrimental cycle that ultimately undermines the integrity of the knowledge system.

In addition, due to limitations in available teaching staff and venues, experimental teaching is mostly based on basic experimental operations and confirmatory experiments, with a relatively small proportion of exploratory experiments. The development of small-class teaching can not only expand the width and breadth of classroom teaching but also enhance the interest of experimental teaching and increase the weight of exploratory experiments [12,15]. In particular, many professional courses in veterinary science are research-oriented and at the cutting edge of knowledge. Take veterinary immunology as an example; immunology knowledge points are updated very frequently, far exceeding the speed of book updates. At the same time, immunology is closely related to daily life, such as COVID-19, seasonal epidemics, allergies, and so on. In light of this situation, from an immunological perspective, it is essential to utilize a solid foundation in basic immunological knowledge to effectively guide students in applying their understanding through practical or experimental training. This approach will enable students to gain a deeper comprehension of and the ability to address immunity-related issues in daily life. It is worth constantly exploring issues related to learning and maintaining personal health, as well as that of students’ families, and even the animals or pets they may care for. During these daily activities, students are encouraged to take initiative by posing questions, devising plans, conducting experiments, and sharing their findings. By constantly increasing their engagement through exploratory experimental teaching, students can foster a spirit of inquiry and cultivate their capacity for independent thinking. Through exploratory experimental training, students can not only broaden their horizons but also be motivated to actively pursue research goals, identify their areas of interest within scientific research, and experience the excitement of discovery. This process helps foster innovative talents with intrinsic motivation [4,24]. Therefore, small-class teaching is very beneficial to the advancement of veterinary science by enhancing learning outcomes and honing professional skills.

In addition, the implementation of small-class teaching in research-oriented universities or comprehensive universities offers significant advantages [1,8]. Taking the subject of veterinary medicine at our school as an example, due to sufficient teaching staff and each teacher specializing in different research fields, targeted instruction can be provided. This allows students to quickly acquire cutting-edge knowledge and master the development of the subject. Simultaneously, it also fulfills the purpose of teaching students according to their aptitude and providing inspiring instruction.

Even with challenges such as a high student population, increased class sizes, uneven teacher distribution, and varying levels of knowledge acquisition among students in different classes, these issues can be addressed through the implementation of the “second classroom”. By establishing case-based and question-based learning [25], students are encouraged to seek information independently, utilize their free time to reinforce and enhance their understanding of learned concepts, preview new material ahead of time, come to class prepared with questions, and ultimately improve their efficiency in acquiring knowledge during lectures. Additionally, tools such as the “rain classroom” have been developed as a new teaching instrument through a joint effort by Xuetang X and Tsinghua University. This tool covers both online and offline teaching scenarios and is fairly easy to use, requiring only PowerPoint and WeChat [26]. It provides teachers with real-time feedback on classroom content, enabling them to promptly address student inquiries and adjust teaching pace and depth based on the students’ grasp of key concepts. Furthermore, research-based reports on specialized topics and situational teaching models—combined with peer evaluations and teacher feedback—significantly enrich the scope and depth of instruction while achieving the goals of widespread knowledge dissemination and problem-solving.

In addition, a multi-level teaching approach can be implemented to provide advanced students with the opportunity to acquire new knowledge and practice new skills, while also allowing average students ample time to comprehend the material. This enables all students to achieve established learning goals through their own efforts. Layered teaching minimizes students’ frustration, maximizes enthusiasm for learning, and helps construct a comprehensive knowledge system by creating targeted learning situations. This allows students to track their individual progress, identify strengths and weaknesses, and employ reasonable and effective measures or methods to complete their studies [27]. For institutions facing an imbalance in their teacher–student ratio, hiring graduate teaching assistants can help alleviate the shortage of teachers. For example, the West China School of Medicine/West China Hospital of Sichuan University has developed a comprehensive evaluation and training system for graduate teaching assistants. This not only meets current teaching needs and addresses teacher shortages but also allows graduate teaching assistants to strengthen their professional knowledge and skills in auxiliary teaching while building a reserve of future educators [28].

## 5. Conclusions

Teaching is a process of interactive communication and learning. By employing diverse teaching modes and methods, educators can foster students’ initiative, cultivate their sense of ownership in facing challenges and fostering innovation, and guide them to collaborate on educational activities, thereby achieving greater results with less effort. The introduction of the “small-class teaching” approach in undergraduate education represents a beneficial endeavor to nurture innovative talents that meet the demands of contemporary society and the nation. This initiative has garnered significant positive feedback.

Small-class teaching allows for full respect of individual differences, enhances the development of students’ critical thinking, expression, and communication skills, as well as improves learning efficiency and effectiveness. Particularly in research or experimental subjects, it enables students to apply theoretical knowledge to practice, facilitating a deeper understanding while enhancing practical abilities such as observation skills, critical thinking capabilities, and hands-on experience—all laying the groundwork for innovation and creativity. However, there are also challenges associated with this approach. For instance, some undergraduates may struggle with motivation or adapting to independent learning while keeping pace with the curriculum’s progression. Additionally, some instructors may continue to employ traditional large-class teaching methods within small-class settings without fully embracing necessary pedagogical reforms. Furthermore, there is a need for further exploration regarding how to select appropriate courses and instructors for implementing small-class teaching reform, how to establish comprehensive evaluation systems, how to develop effective policies supporting academic management departments, and how to enhance schools’ and teachers’ enthusiasm towards investing in small-class teaching reform. These issues require ongoing attention throughout future educational processes.

## Figures and Tables

**Table 1:** Curriculum for veterinary medicine (using animal medicine as an example).

Course Name	Course Category	Whether There Are Experimental Courses
Major Introduction	Foundational Courses	No
Animal Anatomy	Foundational Courses	Yes
Zoology	Foundational Courses	Yes
Histology and Embryology of Animals	Foundational Courses	Yes
Animal Biochemistry	Foundational Courses	Yes
Animal Physiology	Foundational Courses	Yes
Animal Pathophysiology	Foundational Courses	Yes
Traditional Chinese Veterinary Medicine	Professional Core Courses	Yes
Laboratory Animal Science	Professional Core Courses	Yes
Veterinary Lemology	Professional Core Courses	Yes
Veterinary Clinical Diagnosis	Professional Core Courses	Yes
Veterinary Obstetrics	Professional Core Courses	Yes
Veterinary Parasitology	Professional Core Courses	Yes
Veterinary Public Hygiene	Professional Core Courses	No
Physical Test and Chemical Analysis of Foods Derived from Animals	Professional Core Courses	Yes
Veterinary Regulation	Professional Core Courses	No
Microbiological Examination of Animal Food	Professional Core Courses	Yes
Veterinary Microbiology	Professional Core Courses	Yes
Veterinary Immunology	Professional Core Courses	Yes
Veterinary Internal Medicine	Professional Core Courses	Yes
Anatomopathology of Animals	Professional Core Courses	Yes
Veterinary Pharmacology	Professional Core Courses	Yes
Microbiological Inspection of Animal-Derived Foods	Professional Core Courses	Yes
Nutritional, Metabolic, and Poisoning Diseases in Animals	Professional Elective Courses	No
Veterinary Toxicology	Professional Elective Courses	No
Animal Nutrition	Professional Elective Courses	No
Literature Retrieval and Scientific Paper Writing	Professional Elective Courses	No
Veterinary Pharmaceutics	Professional Elective Courses	No
Veterinary Clinical Therapeutics	Professional Elective Courses	No
Veterinary Clinical Differential Diagnosis	Professional Elective Courses	No
Animal Ethology	Professional Elective Courses	No
Small Animal Surgery	Professional Elective Courses	Yes
Veterinary Surgery	Professional Elective Courses	Yes
Veterinary Diagnostic Imaging	Professional Elective Courses	Yes
Animal Experiment Technique	Professional Elective Courses	Yes
Small Animal Ophthalmology	Professional Elective Courses	Yes
Laboratory Animal Medicine	Professional Elective Courses	Yes
Veterinary Epidemiology	Professional Elective Courses	No
Zootechnics	Professional Elective Courses	No
Diseases of Small Animals	Professional Elective Courses	No
Biostatistics and Experimental Design	Professional Elective Courses	No
English for Veterinary Medicine	Professional Elective Courses	No
Veterinary Biologics and Biopharmacy	Professional Elective Courses	No
Wildlife Diseases	Professional Elective Courses	No
Livestock Environmental Hygiene	Professional Elective Courses	No
Traditional Chinese Medicine for Prevention and Treatment of Animal Patterns and Disease	Professional Elective Courses	Yes
Diagnosis of Small Animals CT and MRI	Professional Elective Courses	Yes
Veterinary Clinical Pathology	Professional Elective Courses	Yes
Animal Genetics	Professional Elective Courses	Yes
Chinese Veterinary Acupuncture and Moxibustion	Professional Elective Courses	Yes
Cell and Molecular Biology	Professional Elective Courses	Yes
An Introduction to Animal Protection (The International Curriculum Program)	Cross Open Courses	No
Clinical Practice	Production practice	-
Scientific Research Training	Cognitive practice	-
Training of Innovative Thinking	Innovation and Entrepreneurship Courses	No
Market Quarantine Practice	Production practice	-
Graduation Thesis	-	Yes

## Data Availability

All data contained within this manuscript.
